# Detection and prediction of Alzheimer's and related dementia using administrative data

**DOI:** 10.1002/alz70855_101257

**Published:** 2025-12-23

**Authors:** Korin Reid

**Affiliations:** ^1^ Ellison Laboratories, Chicago, IL, USA

## Abstract

**Background:**

There are more than 5.8 million Americans who suffer from Alzheimer's disease 1. By 2060 13.8 million Americans will be living with Alzheimer's Dementia 2. As of 2022, the financial burden of Alzheimer's Dementia is $321 billion and is expected to reach $1 trillion. 2 Detection and prediction of Alzheimer's and related dementia using widely available data such as electronic health record and electronic claim data could help identify populations at risk for development the disease.

**Method:**

We used electronic health record and claim data to predict Alzheimer's and Related Dementias (ADRD) up to 10 years in advance using electronic health record data from the Mimic‐IV dataset, and electronic claim data form a 5% sample of medicare data from various care settings. For this modeling effort, we used machine learning methods. We also explored techniques to ascertain that models are explainable and work effectively across demographic gorups.

**Result:**

Using the Mimic‐IV dataset, we achieved an AUC of 0.92 predict ADRD up to 10 years in advance. Using a 5% sample of Medicare claim data from diverse healthcare settings, we have achieved an Area Under the Curve (AUC) of 0.86, 0.85, and 0.83 respectively for predicting ADRD 1,3, and 5 years in advance respectively.

**Conclusion:**

Machine learning methods can be utilized to predict ADRD using widely available administrative data such as electronic health record and electronic claim data.

